# Complete genome sequence of *Bacillus licheniformis* HB022, an efficient aflatoxin-degrading bacterial strain

**DOI:** 10.1128/mra.00188-26

**Published:** 2026-05-18

**Authors:** Shubo Li, Si Chen, Shencai Hu

**Affiliations:** 1 School of Life Science and Technology, Wuhan Polytechnic University 74615, Wuhan, China; University of Manitoba, Winnipeg, Canada

**Keywords:** aflatoxin, fermented food, *Bacillus licheniformis*, nanopore

## Abstract

The genome of *Bacillus licheniformis* HB022, which was isolated from Daqu, a Chinese liquor starter, is 4,369,776 bp long with 4,501 protein-coding genes and a GC ratio of 46.2%. Keyword-based filtering from the functional annotations led to the identification of 359 potential aflatoxin-degrading genes.

## ANNOUNCEMENT

Aflatoxins are primarily a group of toxic metabolites produced by mold strains (1, 2). Aflatoxins pose a severe threat to the safety of the food and result in substantial economic losses annually (3, 4). Microbial degradation of aflatoxins is mainly through the disruption of the toxic structural sites of aflatoxin by enzymes, including laccase, horseradish peroxidase, manganese peroxidase, etc. (5, 6), though the detailed mechanisms still remain unclarified.

To screen aflatoxin-degrading strains, different food fermentation raw materials or fermented products were sampled. Finally, a *Bacillus licheniformis* HB022 with high aflatoxin-degrading capability was screened out. The strain originated from a Daqu sample, which was a type of Chinese liquor starter at Yellow Crane Tower Distillery Co., Ltd. (30.55°N, 114.27°E).

DNA was extracted using the QIAGEN Genomic-tip Kit and randomly fragmented with G-TUBE (Covaris, USA) after quality control. The DNA fragments were then subjected to damage repair (NEBNext F PE DNA Repair Mix), end repair, and the addition of polyA at 3′-end (NEBNext Ultra End Repair/dA-Tailing Module). Addition of barcode sequences and adapters was done using Nanopore EXP-NBD104 Kit. Ligation was performed with Nanopore SQK-LSK109 Ligation Sequencing Kit 1D (PM). Then, samples were purified and quantified using Qubit fluorometer from Thermo Fisher Scientific Incorporation. The concentration of the library was greater than 8 ng/μL. Libraries were then sequenced on the PromethION platform (Oxford Nanopore Technologies) using a single R9.4 FLO-MIN106 flow cell for 48 h. Data were acquired in real-time via MinKNOW software and stored as unprocessed fast5 files. Basecalling was conducted using Guppy software (v3.2.6). We got a total of 134,095 raw reads with N50 of 24,066 bp. Adapters, short reads (<2 kb), and low-quality reads (quality score < 6) were removed, leading to a total of 94,473 reads with N50 of 24,855 bp. Reads were assembled using Canu (v1.5) and wtdbg2 (v2.2) (7), followed by CheckM (v1.1.6) analysis to evaluate the genome completeness. Circlator (v1.5.5) was used to circularize the genome. Coding genes were predicted using Prodigal (v2.6.3) (8). Pseudogenes and putative candidates were identified using GeneWise (v2.2.0) (9). tRNA genes were annotated with tRNAscan-SE (v2.0) (10), while rRNA and other non-coding RNA genes were predicted using Infernal (v1.1.3) (11). Default values of the parameters were used for the software mentioned above. The predicted proteins were subjected to BLASTp searches (*e*-value < 1*e*−5) against the National Center for Biotechnology Information (NCBI) Nr, Kyoto Encyclopedia of Genes and Genomes (KEGG), Pfam, and COG/eggNOG databases and Blast2GO against GO for functional annotation. Pathogenicity and antimicrobial resistance determinants were annotated by BLASTp against the databases of CAZy, TCDB, CARD, and VFDB (12–15).

With a coverage of 300×, the reads were assembled into a contig of 4,369,776 bp, which is a complete genome based on CheckM results. The genome was able to be circularized by Circlator (v1.5.5) with the parameter “minimus2 --no_pre_merge.” The start position of the genome is the gene nearest to the center of the contig. We summarized the results of assembly and annotation in Table 1.

**TABLE 1:** Genome assembly and annotation of *Bacillus licheniformis* HB022

Parameter	Value
Assembly	
Genome length (bp)	4,369,776
Raw reads	134,095
Reads	94,473
Contig	1
GC content (%)	46.2
Annotation	
Protein-coding genes	4,501
rRNAs	24
tRNAs	84
ncRNAs	101
Repeat elements	105
CRISPRs	5
Pseudogenes	3
Genomic islands	11

## Data Availability

The complete chromosome sequence of *Bacillus licheniformis* HB022 has been submitted to GenBank with accession number JBVQOM000000000 under BioProject number PRJNA1429879 (BioSample accession number SAMN56256788 and SRA accession number SRR37482908). The supplementary data, including the annotations of genes and the list of the potential aflatoxin-degrading genes, have been deposited in Figshare with DOI https://doi.org/10.6084/m9.figshare.31557130.
